# Do neural nets learn statistical laws behind natural language?

**DOI:** 10.1371/journal.pone.0189326

**Published:** 2017-12-29

**Authors:** Shuntaro Takahashi, Kumiko Tanaka-Ishii

**Affiliations:** 1 The University of Tokyo, Graduate School of Frontier Sciences, Chiba 277-8563, Japan; 2 The University of Tokyo, Research Center for Advanced Science and Technology, Tokyo 153-8904, Japan; Universidad Veracruzana, MEXICO

## Abstract

The performance of deep learning in natural language processing has been spectacular, but the reasons for this success remain unclear because of the inherent complexity of deep learning. This paper provides empirical evidence of its effectiveness and of a limitation of neural networks for language engineering. Precisely, we demonstrate that a neural language model based on long short-term memory (LSTM) effectively reproduces Zipf’s law and Heaps’ law, two representative statistical properties underlying natural language. We discuss the quality of reproducibility and the emergence of Zipf’s law and Heaps’ law as training progresses. We also point out that the neural language model has a limitation in reproducing long-range correlation, another statistical property of natural language. This understanding could provide a direction for improving the architectures of neural networks.

## 1 Introduction

Deep learning has performed spectacularly in various natural language processing tasks such as machine translation [1], text summarization [2], dialogue systems [3], and question answering [4]. A fundamental question that we ask, however, is why deep learning is such an effective approach for natural language processing. In contrast to the progress made in applying deep learning, our understanding of the reasons for its effectiveness remains limited because of its inherent complexity.

One approach to tackling this problem is mathematical analysis of the potential of neural networks [5–11]. Here, we take a different empirical approach based on the statistical properties of text generated by neural networks. Precisely, we compare the statistical properties of pseudo-text generated by a neural language model with those of the real text with which the model is trained.

We have found that two well acknowledged statistical laws of natural language—Zipf’s law [12] and Heaps’ law [13] [14] [15]—almost hold for the pseudo-text generated by a neural language model. This finding is notable because previous language models, such as Markov models, cannot reproduce such properties, and mathematical models, which are designed to reproduce statistical laws [16] [17], are also limited in their purpose. As compared with those models, neural language models are far more advanced in satisfying the statistical laws. We find a shortcoming of neural language models, however, in that the generated pseudo-text has a limitation with respect to satisfying a third statistical property, the long-range correlation. The analyses described in this paper contribute to our understanding of the performance of neural networks and provide guidance as to how we can improve models.

## 2 Neural language models generate text following Zipf’s law and Heaps’ law

### 2.1 Neural language model

We constructed a neural language model that learns from a corpus and generates a pseudo-text, and then investigated whether the model produced any statistical laws of language. The language model estimates the probability of the next element of the sequence, *w*_*i*+1_, given its past sequence or a subset as context:

$$\begin{array}{c} P(w_{i+1}|w_{i-k}^{i}), \end{array}$$ (1)

where *k* is the context length, and $w_{i}^{j}$ is the subsequence of text between the *i*th and *j*th elements. Bengio et al. [18] first proposed the concept of a neural language model, and this concept has been explored mainly with recurrent neural networks (RNNs) [19] [20] [21]. We construct a language model at the character level, which we denote as a stacked long short-term memory (LSTM) [22] model. This model consists of three LSTM layers with 256 units each and a softmax output layer. We treat this stacked LSTM model as a representative of neural language models.

In all experiments in this article, the model was trained to minimize the cross-entropy by using an Adam optimizer with the proposed hyper-parameters [23]. The context length *k* was set to 128. To avoid sample biases and hence increase the generalization performance, the dataset was shuffled during the training procedure: i.e. every one learning scan of the training data is conducted in a different shuffled order. This is a standard configuration with respect to previous research on neural language models [19] [20] [21] [24].

In the normal scheme of deep learning research, the model learns from all the samples of the training dataset once during an epoch. In this work, however, we redefined the scheme so that the model learns from 1% of the training dataset during every epoch. That is, the model learns from all the samples every 100 epochs. We adopted this definition because the evolutions of Zipf’s law and Heaps’ law are so fast that their corresponding behaviors are clearly present after the model has learned from all the samples once. Although we discuss this topic in Section 3, we emphasize here that either this redefinition or some other approach was necessary to observe the model’s development with respect to Zipf’s law and Heaps’ law.

Generation of a pseudo-text begins with 128 characters in succession as context, where the 128-character sequence exists in the original text. One character to follow the context is chosen randomly according to the probability distribution of the neural model’s output. The context is then shifted ahead by one character to include the latest character. This procedure is repeated to produce a pseudo-text of 2 million characters unless otherwise noted. The following is an example of a generated pseudo-text: *“and you gracious inherites and what saist i should agge the guest.”*

We chose a character-level language model because word-level models have the critical problem of being unable to introduce new words during generation: by definition, they do not generate new words unless special architectures are added. A word-level model typically processes all words with rarity above a certain threshold by transforming each into a singular symbol “unk”. With such a model, there is a definite vocabulary size limit, thus destroying the tail of the rank-frequency distribution. Zipf’s law and Heaps’ law therefore cannot be reproduced with such a model. There have been discussions and proposals regarding this “unk” problem [25] [26], but there is no de facto standard approach, and the problem is not straightforward to solve. Therefore, we chose a character-level language model.

Note that the English datasets, consisting of the Complete Works of Shakespeare and The Wall Street Journal (WSJ), were preprocessed according to [19] by making all alphabetical characters lower case and removing all non-alphabetical characters except spaces. Consecutive spaces were also reduced to one space.

### 2.2 Zipf’s law and Heaps’ law for pseudo-texts generated by neural language models

Zipf’s law and Heaps’ law are two representative statistical properties of natural language. Zipf’s law states that, given word rank *u* and frequency *F*(*u*) for a word of rank *u*, the following proportionality holds:

$$\begin{array}{c} F\left(u\right)\propto u^{-\xi }.\;\; \end{array}$$ (2)

This exponent *ξ* is approximately 1.0, according to Zipf, for individual word occurrence (uni-grams), but, as will be shown, a power law with smaller *ξ* values holds for longer word sequences (i.e., *n*-grams, including 2-grams, 3-grams, and so on).

Heaps’ law, another statistical law of natural language, underlies the growth rate of vocabulary size (the number of types) with respect to text length (the number of tokens). Given vocabulary size *V*(*m*) for a text of length *m*, Heaps’ law indicates that

$$\begin{array}{c} V\left(m\right)\propto m^{\zeta }. \end{array}$$ (3)

The power law underlying vocabulary growth was reported even before Heaps’ paper [13], as in [14] [15], but in this paper we refer to the law as Heaps’ law. Zipf’s law and Heaps law are known to have a theoretical relationship, as discussed in [27] [28] [29].

**Fig 1:**
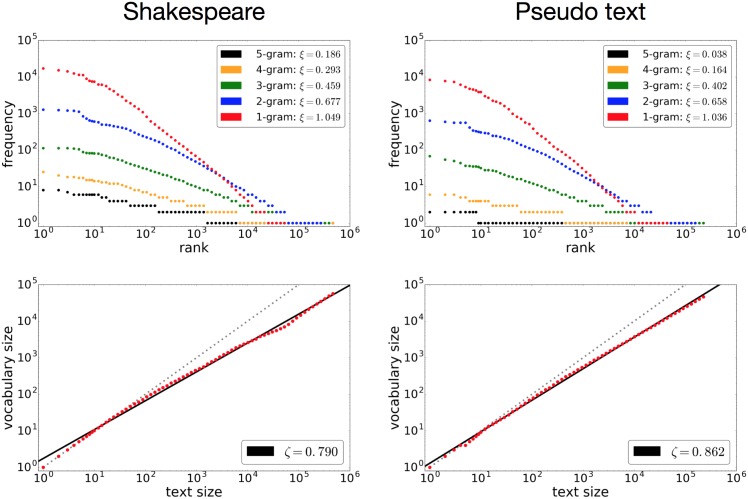
The rank-frequency distribution and vocabulary growth of the Complete Works of Shakespeare (left) and the corresponding pseudo-text generated by the stacked LSTM model (right). All axes in this and subsequent figures in this paper are in logarithmic scale, and the plots were generated using logarithmic bins. The model learned from 4,121,423 characters in the Complete Works of Shakespeare, which was preprocessed as described in the main text. The colored sets of the plots of the figures in the first row show the rank-frequency distributions of 1,2,3,4,5 grams. The figures in the second row show the vocabulary growth in red. The exponents *ξ* and *ζ* (defined in formulas (2) and (3), respectively) were estimated by linear regression from in log-log scale. For all graphs, the corresponding estimated exponents are indicated in the caption, and the black solid line in each vocabulary growth figure shows the fitted line. The dashed line indicates a reference with an exponent of 1.0. The same applies to all other rank-frequency distribution and vocabulary growth plots in this paper.

The upper-left graph in Fig 1 shows the rank-frequency distribution of the Complete Works of Shakespeare, of 4,121,423 characters, for *n*-grams ranging from uni-grams to 5-grams. As Zipf stated, the uni-gram distribution approximately follows a power law with an exponent of 1.0. The higher *n*-gram distributions also follow power laws but with smaller exponents. Note that intersection of the uni-gram and 2-gram distributions in the tail is typically observed for natural language. The lower-left graph in Fig 1 shows the vocabulary growth of the Complete Works of Shakespeare. The red points show the vocabulary size *V*(*m*) for every text length *m*, and the exponent *ζ* was estimated as 0.773, as shown by the black fitting line. This exponent is larger than that reported in previous works, and this was due to the preprocessing, as previously mentioned.

The graphs on the right side of Fig 1 show the corresponding rank-frequency distribution and vocabulary growth of the pseudo-text generated by the stacked LSTM. The rank-frequency distribution is almost identical to that of the Complete Works of Shakespeare for uni-grams and 2-grams, reproducing the original shape of the distribution. The distributions for longer *n*-grams are also well reproduced. As for the vocabulary growth, the language model introduces new words according to a power law with a slightly larger exponent than that of the original text. This suggests a limitation on the recognition of words and the organization of *n*-gram sequences. These results indicate that the stacked LSTM can reproduce an *n*-gram structure closely resembling the original structure.

**Fig 2:**
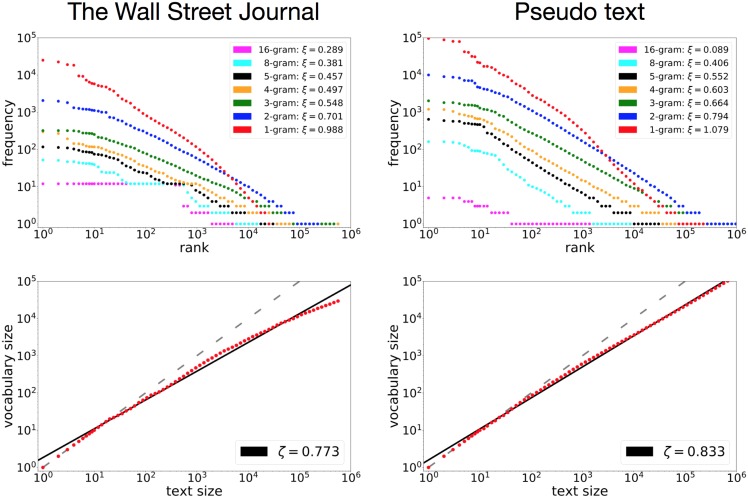
The rank-frequency distribution and vocabulary growth of The Wall Street Journal (left) and the corresponding pseudo-text generated by the stacked LSTM model (right). The model learned from 4,780,916 characters. The length of the pseudo-text is 20 million characters. The rank-frequency distributions are shown for 1,2,3,4,5,8,16-grams. The preprocessing procedure was the same as for the Complete Works of Shakespeare.

**Fig 3:**
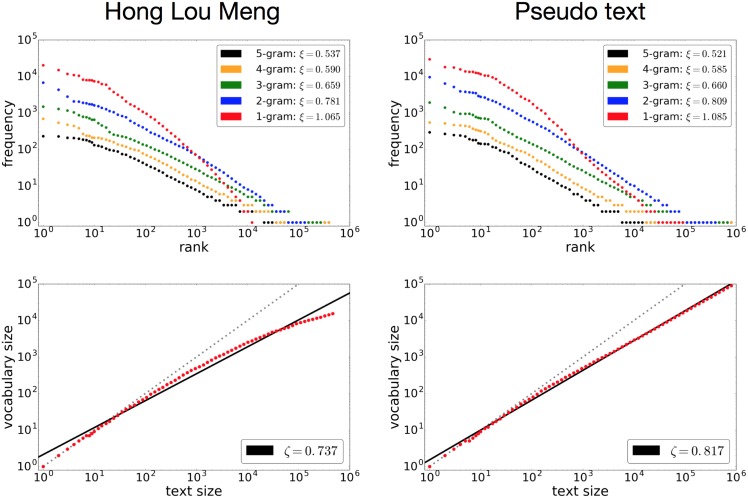
The rank-frequency distribution and vocabulary growth of the Chinese literary work Hong Lou Meng, consisting of 2,932,451 bytes (left), and the pseudo-text generated by the stacked LSTM (right). The text was processed at the byte level with word borders.

The potential of the stacked LSTM is still apparent even when we change the kind of text. Figs 2 and 3 show results obtained using The Wall Street Journal (from the Penn Tree Bank Dataset) and a Chinese literary text, Hong Lou Meng by X. C. Xueqin, respectively, and the corresponding pseudo-texts generated by the stacked LSTM. The WSJ text of 4,780,916 characters was subjected to the same preprocessing as for the Complete Works of Shakespeare. To deal with the large vocabulary size of the Chinese characters, the model was trained at the byte level [30] for Hong Lou Meng, resulting in a text of 2,932,451 bytes. To measure the rank-frequency distribution and vocabulary growth at the word level, the model had to learn not only the sequence of bytes but also the splits between them.

The observations made for the Complete Works of Shakespeare apply also to Figs 2 and 3. We observe power laws for both the rank-frequency distributions and the vocabulary growth. The stacked LSTM replicates the power-law behaviors well, reproducing approximately the same shapes for smaller *n*-grams. The intersection of the uni-gram and 2-gram rank-frequency distributions is reproduced as well. As for the vocabulary growth, the reproduced exponents were a little larger than the original values, as seen for the case of Shakespeare.

Fig 2 also highlights the high capacity of the stacked LSTM in learning with long *n*-grams. The top right graph demonstrates that the stacked LSTM could repeat the same expression of 8-grams and 16-grams obeying Zipf’s law. In the Complete Works of Shakespeare, written by a single author, long repeated *n*-grams hardly occur, but the WSJ dataset contains many of these. For the WSJ data, the rank-frequency distributions of 8- and 16-grams do not obviously follow power laws, mainly because of repetition of the same expressions. With such a corpus, the stacked LSTM can also reproduce the power-law behavior of the rank-frequency distribution of long *n*-grams.

These results indicate that a neural language model can learn the statistical laws behind natural language, and that the stacked LSTM is especially capable of reproducing both patterns of *n*-grams and the properties of vocabulary growth.

We also tested language models with different architectures. S1 Fig shows results with different neural architectures for pseudo-texts generated for the Complete Works of Shakespeare: a convolutional neural net (CNN), simple RNN, single-layer LSTM, and stacked LSTM. The stacked LSTM model was explained in Section 2.1, while the details of the other models are given in the caption of S1 Fig. The two bottom right graphs for the stacked LSTM are identical to the two righthand graphs in Fig 1. Overall, all the models using an RNN reproduce power law behavior, but a closer look reveals greater capacity with the stacked LSTM. With the CNN (upper left), on the other hand, the shape of the rank-frequency distribution is quite different, and the exponent of the vocabulary growth is too large. The simple RNN (upper right) shows weaker capacity in reproducing longer *n*-grams, and the exponent is still too large. Finally, the single-layer LSTM (bottom left) is less capable of learning the longest *n*-gram of 5-grams as compared with the stacked LSTM (bottom right).

## 3 The Emergence of Zipf’s law and Heaps’ law

**Fig 4:**
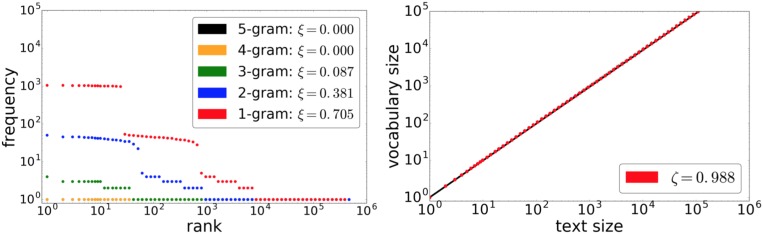
Rank-frequency distribution (left) and vocabulary growth (right) for a text of 20 million characters generated by the stacked LSTM without learning.

The stacked LSTM acquires the behaviors of Zipf’s law and Heaps’ law as learning progresses. It starts learning obviously at the level of a monkey typing. Fig 4 shows the rank-frequency distribution and vocabulary growth of a texts generated by the stacked LSTM without training. Each case from uni-grams to 3-grams roughly forms a power-law kind of step function. The vocabulary growth follows a power law with exponent *ζ* ≈ 1 because monkey typing consistently generates “new words.”

As shown by Fig 4, monkey-typed texts can theoretically produce power-law-like behaviors in the rank-frequency distribution and vocabulary growth. [31] demonstrates how monkey typing generates a power-law rank-frequency distribution. Following the explanation in [32], we briefly summarize the rationale as follows. Consider a monkey that randomly types any of *n* characters and the space bar. Since a space separates words, let its probability be *q*, and then each of the other characters is hit uniformly with a probability of (1 − *q*)/*n*. Given that the number of words of length *c* is *n*^*c*^, and that longer words are less likely to occur, then the rank frequency of a word of length *c* is between *S*(*c*) + 1 and *S*(*c* + 1), where $S\left(c\right)=\sum_{i=1}^{c}n^{i}$. Since $S\left(c\right)=\frac{n^{c}-1}{n-1}$, the rank *r*_*c*_ of a word of length *c* grows exponentially with respect to *c*; i.e., *r*_*c*_ ≈ *n*^*c*^. Given that the probability of occurrence of a word of length *c* is $q{\left(\frac{1-q}{n}\right)}^{c}$, by replacing *c* with the rank, we obtain the rank-probability distribution as

$$\begin{array}{c} P\left(r_{c}\right)=q{\left(\frac{1-q}{n}\right)}^{\log\;r_{c}}=q{\left(r_{c}\right)}^{\log(1-q)-1}, \end{array}$$ (4)

where the log is taken with base *n*. This result shows that the probability distribution follows a power law with respect to the rank. The LSTM models therefore start learning by innately possessing a power-law feature for the rank-frequency distribution and vocabulary growth. The learning process thus smooths the step-like function into a more continuous distribution; moreover, it decreases the exponent for vocabulary growth. [33] reports empirically that when the probabilities of each character are different, the rank-frequency distribution becomes smoother. While learning progresses, the exponent *ζ* is lowered by learning patterns within texts.

**Fig 5:**
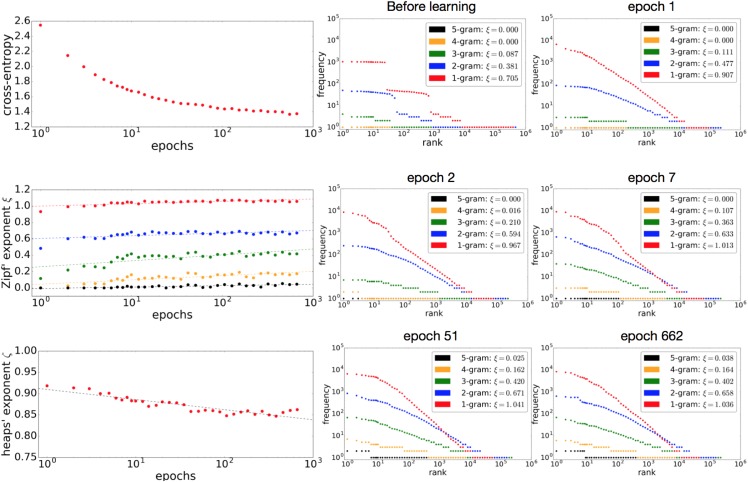
Training cross entropy as a function of the number of epochs (upper left); Zipf’s exponent *ξ* (middle left) and Heaps’ exponent *ζ* (lower left) of pseudo-texts generated at different epochs; and the rank-frequency distributions of the pseudo-texts at various epochs (right) for the Complete Works of Shakespeare. The left-hand graphs are in logarithmic scale for the x-axes and linear scale for the y-axes. The fitting lines for Zipf’s exponent *ξ* and Heaps’ exponent *ζ* were estimated from a linear regression with a semi-log scale.

Fig 5 illustrates the training progress of the language model for the Complete Works of Shakespeare. The upper-left graph shows the cross entropy of the model at different training epochs. The training successfully decreases the cross entropy and reaches a convergent state.

The middle and lower left graphs in Fig 5 show the Zipf’s exponent *ξ* and Heaps’ exponent *ζ* of the pseudo-texts generated at different epochs. At the very beginning of training, the Zipf’s exponents *ξ* tend to be smaller than that of the original dataset. They generally increase and become equivalent to the values of the original datasets for short *n*-grams or remain at smaller values for long *n*-grams. As the model minimizes the cross entropy, the Heaps’ exponent *ζ* generally decreases, with some fluctuation, by learning words. It roughly stops decreasing, however, at around 10^2^ to10^3^ epochs. The fact that the exponents of Heaps’ law cannot reach the value of the original text indicates some limitation in learning.

The right-hand side of Fig 5 shows the rank-frequency distributions of the pseudo-texts generated at different epochs. The stacked LSTM model reproduces the power-law behavior well for uni-grams and 2-grams, and partially for 3-grams, with just a single epoch (upper right). Such behavior for 4-grams appears in epoch 2 (middle left), and the intersection of the uni-gram and 3-gram power laws appears in epoch 7 (middle right). Power-law behavior for 5-grams emerges in epoch 51 (bottom left), and no further qualitative change is observed afterwards (bottom right).

As training progresses, the stacked LSTM first learns short patterns (uni-grams and 2-grams) and then gradually acquires longer patterns (3- to 5-grams). It also learns vocabulary as training progresses, which lowers the exponent of Heaps’ law. There are no tipping points at which the neural nets drastically change their behavior, and the two power laws are both acquired at a fairly early stage of learning.

## 4 Neural language models are limited in reproducing long-range correlation

Natural language has structural features other than *n*-grams that underlie the arrangement of words. A representative of such features is grammar, which has been described in various ways in the linguistics domain. The structure underlying the arrangement of words has been reported to be scale-free, globally ranging across sentences and at the whole-text level. One quantification methodology for such global structure is long-range correlation.

Long-range correlation describes a property by which two subsequences within a sequence remain similar even with a long distance between them. Typically, such sequences have a power-law relationship between the distance and the similarity. This statistical property is observed for various sequences in complex systems. Various studies [34–41] report that natural language has long-range correlation as well.

### 4.1 The power decay of mutual information is unlikely to hold for natural language text

Measurement of long-range correlation is not a simple problem, as we will see, and various methods have been proposed. [24] proposes applying mutual information to measure long-range dependence between symbols. The mutual information at a distance *s* is defined as

$$\begin{array}{c} I_{s}\left(X,Y\right)=\sum_{X=a,Y=b}P\left(X,Y\right)\log\frac{P(X,Y)}{P(X)P(Y)} \end{array}$$ (5)

where *X* and *Y* are random variables of elements in each of two subsequences at distance *s*.

**Fig 6:**
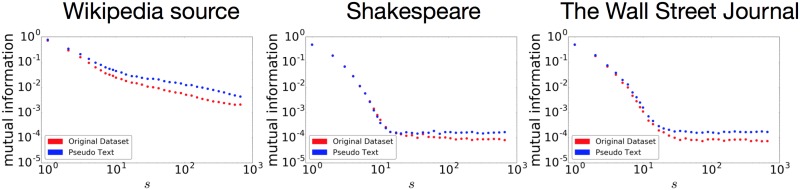
Mutual information for a Wikipedia source (left), the Complete Works of Shakespeare (middle), and The Wall Street Journal (right). The red and blue points represent the mutual information of the datasets and the generated pseudo-texts, respectively. Following [24], the Wikipedia *source* was preprocessed to exclude rare symbols.

[24] proves mathematically how a sequence generated with their simple recursive grammar model results in power decay of the mutual information. They also provide empirical evidence that a Wikipedia source from the enwik8 dataset exhibits power decay of the mutual information, and that a pseudo-text generated from Wikipedia also exhibits power decay when measured at the character level. The left graph in Fig 6 shows our reproduction of the mutual information for the Wikipedia source used in [24]. For each of the graphs in the figure, the horizontal axis indicates the distance *s*, and the vertical axis indicates the mutual information. The red and blue points represent the results for the real texts and the pseudo-texts, respectively. By using the data in [24], we could reproduce their results: for the Wikipedia source, the mutual information exhibits power decay, and this statistical property was also learned by the stacked LSTM.

We doubt, however, that the power decay of the mutual information is being properly observed for a natural language text when measured with the method proposed in [24]. The middle and right graphs in Fig 6 show the results for the Complete Works of Shakespeare and The Wall Street Journal, which are more standard natural language datasets. The mutual information exhibits an exponential decay showing short-range correlation similar to what they reported as the behavior of Markov models, and it almost reaches a plateau just after a 10-character distance for both datasets. This plateau represents a state in which the probabilistic distributions of *a* and *b* pairs become almost the same because of the low frequency problem. Following the statistical properties of the datasets, the stacked LSTM replicates this exponential decay well. [24] also examines a natural language corpus, corpatext. Unfortunately, corpatext is poorly organized, as it contains a huge number of repeats of long *n*-grams, chains of numbers, many meta-characters, and successive spaces. Our measurement of mutual information with corpatext (which was preprocessed to delete meta-characters and successive spaces) gave an exponential decay up to 10 characters. This observation is similar to the results for the Complete Works of Shakespeare and WSJ. The mutual information slowly decayed after a length of 10, however, which could lead to misinterpretation of the power decay. [24] does not clearly mention any preprocessing or measurement of the mutual information of a pseudo-text for corpatext. We cannot reach a solid conclusion with such an unorganized corpus.

There are two reasons for this difference in results between the Wikipedia source and the Shakespeare/WSJ: the kind of data, and the quantification method. Regarding the kind of data, we must emphasize that [24] does not use any standard natural language text for verification. Instead, they use the wiki *source*, including all Wikipedia annotations. Therefore, Wikipedia is strongly grammatical, that they consider for their mathematical proof.

As for the problem of the quantification method, as seen from the plateau appearing in the results for the Shakespeare and WSJ datasets, the mutual information in its basic form is highly susceptible to the low frequency problem. Therefore, [24] verifies data with a small alphabet size (including DNA sequences). When the alphabet is increased to the size of the Chinese character set, the mutual information reaches the plateau almost immediately; when using words, the plateau is reached in only two or three points.

Still, [24] provides a crucial understanding of neural nets: the stacked LSTM (or other LSTM-like models) can replicate the power decay of the mutual information, if it exists in the original data. Whether such strong long-range correlation exists, however, depends on the data type. Given all other reports, as will be mentioned shortly, long-range correlation *does* exist in natural language texts. The problem of how to quantify it is non-trivial, however, and the mutual information, as proposed, is not always a good measurement for natural language.

### 4.2 Neural language models cannot reproduce the power decay of the autocorrelation function in natural language

Quantification of long-range correlation has been studied in the statistical physics domain and has been effective in analyzing extreme events in natural phenomena and financial markets [42–49]. The long-range correlation in this application is also a scale-free property taking a power-law form. Long-range correlation is explained here as a “clustering phenomenon” of rare events. This could have some relation to an underlying grammar-like structure in a sequence, but the measure might quantify a phenomenon different from that captured by the mutual information.

The application of long-range correlation to natural language is controversial, because all proposed methods are for numerical data, whereas natural language has a different nature. Various reports show how natural language is indeed long-range correlated [34–36, 38–40]. We apply the most recent method [41] to investigate whether the pseudo-text generated by a stacked LSTM retains long-range correlation.

This method is based on the autocorrelation function applied to a sequence *R* = *r*_1_, *r*_2_, …, *r*_*N*_ with mean *μ* and standard deviation *σ*:

$$\begin{array}{c} C\left(s\right)=\frac{1}{\left(N-s\right)\sigma^{2}}\sum_{i=1}^{N-s}\left(r_{i}-\mu \right)\left(r_{i+s}-\mu \right), \end{array}$$ (6)

with *C*(0) = 1.0 by definition. Note that this function *C*(*s*) measures the similarity between two subsequences that are distance *s* apart. If this autocorrelation function exhibits a power decay as follows,

$$\begin{array}{c} C\left(s\right)=C\left(1\right)s^{-\gamma },s>0, \end{array}$$ (7)

then the sequence *R* is long-range correlated. The functional range of *C*(*s*) is between −1.0 and 1.0. If there is no correlation, *C*(*s*) is almost zero; if the sequences are well correlated positively, *C*(*s*) takes a positive value. The method used here is based on the intervals between rare words, and we use the rarest 1/16th of words among all words appearing in a text, following [41], in defining the interval sequence *R*.

**Fig 7:**
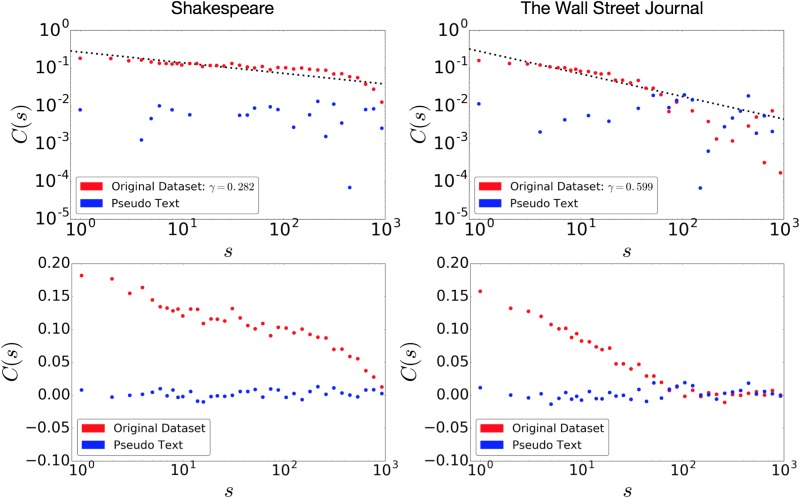
Long-range correlation measured with the autocorrelation function by the method of [41] for the original and generated texts of the Complete Works of Shakespeare (left) and The Wall Street Journal (right). The upper and lower graphs are in log-log and semi-log scale, respectively. The fitting line for The Wall Street Journal was estimated from the data points where *s* < 100.

Fig 7 shows the autocorrelation functions of the Complete Works of Shakespeare (left) and The Wall Street Journal (right) at the word level. The upper graphs show log-log plots, whereas the lower graphs show semi-log plots for the same sets of results. The red and blue points represent the original dataset and the pseudo-text, respectively. For the original Complete Works of Shakespeare, the results exhibit a clear, slow power decay up to *s* = 10^3^. This behavior is similar to that of other literary texts reported in [41]. In contrast, the autocorrelation function of the pseudo-text takes values around 0 for any *s*. The Wall Street Journal results show similar behavior. The power decay is faster than that of the Complete Works of Shakespeare and other single-author literary texts, but the autocorrelation function still takes positive values and exhibits power decay up to about *s* = 10^2^.

In summary, this analysis provides qualitative evidence regarding a shortcoming of the stacked LSTM: it has a limitation with respect to reproducing long-range correlation, as quantified using a method proposed in the statistical physics domain.

To further clarify the stacked LSTM’s performance and limitation, we conducted three experiments from different perspectives. First, S2 Fig shows the encoding rate decay of the original text (WSJ) with different types of shuffling and the pseudo-text. The encoding rate is a measure of a text’s predictability, and we provide more explanation in the caption of S2 Fig. The results show that the pseudo-text generated by the stacked LSTM model is more predictable than the word-shuffled WSJ, and less predictable than both the original and document-shuffled WSJ.

Second, S3 Fig shows the mutual information and autocorrelation function of the original text (Shakespeare) and pseudo-texts generated by different neural architectures. The mutual information decays rapidly up to around *s* = 10, and the models except for the CNN model reproduce the behavior of the mutual information of the original dataset well. On the other hand, the power decay exhibited by the original dataset was never reproduced by any model that we tested.

Third, S4 Fig shows the autocorrelation function for the French novel, Les Misérables, and the text translated into English by a neural machine translation system. We obtained the translated text from the Google Cloud Translation API(https://cloud.google.com/translate/). The translated text maintains the power decay of the autocorrelation function observed for the original text. This can be explained from the properties of machine translation. Machine translation is not expected to radically change the order of corresponding words between sentences. Therefore, as long as the translation system has the capacity to output rare words in the original text, the autocorrelation should be preserved.

Because long-range correlation is a global, scale-free property of a text, one reason for the limitation of the stacked LSTM could lie in the context length of *k* = 128 at the character level. Considering the availability of computational resources, however, this setting was a maximum limit, as the number of layers to be computed substantially increases with the context length. Moreover, it has been empirically reported that an LSTM architecture cannot retain past information beyond a length of 100 [22].

One possible future approach is to test new neural models with enhanced long-memory features, such as a CNN application [50] [51] or the hierarchical structure of an RNN [52] [53]. Overall, the behavior of pseudo-texts with respect to the statistical laws of natural language partly reveals both the effectiveness and limitations of neural networks, which tend to remain black boxes because of their complexity. Analysis using statistical laws could provide a direction towards improving the architectures of neural networks.

## 5 Conclusion

To understand the effectiveness and limitations of deep learning for natural language processing, we empirically analyzed the capacity of neural language models in terms of the statistical laws of natural language. This paper considered three statistical laws of natural language: Zipf’s law, the power law underlying the rank-frequency distribution; Heaps’ law, the power-law increase in vocabulary size with respect to text size; and long-range correlation, which captures the self-similarity underlying natural language sequences.

The analysis revealed that neural language models satisfy Zipf’s law, not only for uni-grams, but also for longer *n*-grams. To the best of our knowledge, this is the first language model that can reproduce a statistical law at such a level. The language models also satisfy Heaps’ law: they generate text with power-law vocabulary growth. The exponent remained higher than for the original texts, however, which showed both the limitation of detecting words and the self-organization of linguistic sequences.

Finally, a stacked LSTM showed a limitation with respect to capturing the long-range correlation of natural language. Investigation of a previous work [24] revealed that if the original learning text has a strong grammatical structure, then a stacked LSTM has the potential to reproduce it. A standard natural language text, however, does not have such a feature. The long-range correlation quantified with another methodology for the original texts was not reproduced by the stacked LSTM.

Our analysis suggests a direction for improving language models, which has always been the central problem in handling natural language on machines. The current neural language models are unable to handle the global structures underlying texts. Because the Zipf’s law behavior with long *n*-grams was reproduced well by the stacked LSTM, this neural language model has high capacity for recognizing and reproducing local patterns or phrases in the original text. The model could not, however, reproduce the long-range correlation measured by the autocorrelation function for intervals between rare words. This long-range correlation cannot be reduced to local patterns but rather is a representation of global structure in natural language. This irreproducibility demonstrates the limitation of neural language models and is a challenge for language modeling with neural networks.

Our future work thus includes exploring conditions to reproduce the long-range correlation in text with language models, including both stochastic and neural language models.

## Supporting information

S1 Fig Comparison of the rank-frequency distribution and vocabulary growth of different models for the Complete Works of Shakespeare. Each pair of graphs consists of the rank-frequency distribution (upper graph) and the vocabulary growth (lower graph). The models had the following specifications. CNN: 8 layers of one-dimensional convolution with 256 filters having a width of 7 without padding and global max pooling after the last convolutional layer. The activation function was rectified linear-unit, and batch normalization was applied before activation in every convolutional layer. Simple-RNN: 1 layer of RNN with 512 units and an output softmax layer. Single-layer LSTM: 1 layer of LSTM with 512 units and an output softmax layer. Stacked-LSTM: as described in Section 2.
(EPS)

S2 FigEncoding rate decay and fitting functions for the WSJ with various shuffling methods and the corresponding pseudo-text generated by the stacked LSTM.Let $X_{1}^{n}$ be a text of length *n* characters, and let $R(X_{1}^{n})$ be its size in bits after compression. Then the code length per unit, i.e., the encoding rate, is defined as $r\left(n\right)=R\left(X_{1}^{n}\right)/n$. The more predictable the text is, the smaller *r*(*n*) becomes; therefore, *r*(*n*) is smaller for longer *n*, exhibiting decay. The fitting function here is a power ansatz function, *f*(*n*) = *An*^*β*−1^ + *h*, proposed by [54], and the compressor was PPMd, using the 7zip application (refer to [55] for details). In addition to the original text, the WSJ was shuffled at the character, word, and document levels. The decay of the pseudo-text is situated between the decays of the word- and document-shuffled versions, indicating clearly that its predictability is situated between the two and suggesting that the pseudo-text has lower predictability as compared to the original and document-shuffled WSJ.(EPS)

S3 Fig Comparison of the mutual information and autocorrelation function for pseudo-texts generated by different models on the Complete Works of Shakespeare. Each pair of graphs represents the mutual information (upper) and the autocorrelation function (lower). The results were obtained with a CNN (upper left), simple RNN (upper right), single-layer LSTM (lower left), and stacked LSTM (bottom right, the same graphs from Fig 1). For the specifications of every model, see the caption of S1 Fig.
(EPS)

S4 Fig Autocorrelation functions of the original text of Les Misérables (V. Hugo, 621,641 words) in French and its corresponding text translated into English by the Google Cloud Translation API https://cloud.google.com/translate/, which is based on neural machine translation. Because of the API’s requirements, the original text was split into 5000 characters to obtain the translated text. Despite the results given in Section 4.2, the translated text exhibits long-range correlation as measured by the autocorrelation function. This result does not contradict our observation in Section 4.2, because translation does not radically change the order of words and the translation system has the capacity to output rare words.
(EPS)
